# Anti‐racist approaches to increase access to general and oral health care during a pandemic in the Pacific Islander community

**DOI:** 10.1111/jphd.12519

**Published:** 2022-06-21

**Authors:** Matthew M. Oishi, Rachelle Robley, Megan K. Inada, Jason Hiramoto

**Affiliations:** ^1^ David R. Breese Center for Community Oral Health Kokua Kalihi Valley Comprehensive Family Services Honolulu Hawaiʻi USA; ^2^ Department of Preventive and Community Dentistry University of Iowa College of Dentistry Lowa City Lowa USA; ^3^ Grants Department Kokua Kalihi Valley Comprehensive Family Services Honolulu Hawaiʻi USA; ^4^ Research Department Kokua Kalihi Valley Comprehensive Family Services Honolulu Hawaiʻi USA

**Keywords:** community dentistry, dental public health, FQHC, Pacific islanders, racism, social determinants of health

## Abstract

Limited data exists on Pacific Islander (PI) health, but a growing body of literature reports the existence of racial discrimination and inequities and mistrust of the healthcare system, leading to poor health outcomes. When COVID‐19 restricted health services, such inequities and mistrust due to historical trauma were magnified. This report describes one federally qualified health center's dental department's response utilizing culture‐based approaches, community relationships, and the social determinants of health (SDOH) to dispel the stigma of COVID and restrictions on in‐person care in order to lower barriers to accessing care. When the dental department transitioned to emergency‐only care, staff were redeployed to address significant inequities facing the PI community. Redeployment activities included building relationships with the most vulnerable patients, delivering healthy foods, supplies, oral hygiene kits to households, and canvasing neighborhood businesses with public health education. The mobile dental clinic, a trusted symbol in the community, also brought public health education to community testing events and food distributions. From March 2020 to July 2020, staff conducted over 800 outreach calls for health and food security, delivered over 2000 care packages and oral hygiene kits. Also, frequent community outreach by the mobile dental clinic led to a 10‐fold increase in COVID testing. Investing in relationship building can maintain access to health care and build trust in the health care system for PI communities. This approach may be relevant to others serving other communities experiencing racism.

Pacific Island immigrants (PI) are a rapidly growing ethnic group in Hawaiʻi and also the continental United States (US). In addition to those from Polynesia (and Melanesia to some extent), many PIs in Hawaiʻi and the US come from the Federated States of Micronesia, one of the three nations with Compacts of Free Association (COFA) with the US. These compacts were a result of the United States' involvement in Micronesia during the post‐World War II period and allows COFA citizens free ability to enter and work in the US, and access education and health services.^1^ Until 2021, COFA citizens were not able to fully participate in Medicaid, despite a history of nuclear testing and militarization by the US,^2^, ^3^, ^4^ resulting in a high prevalence of chronic diseases as well as generational trauma and distrust of the US and western systems of care.^5^

Limited data on the general and oral health of PIs suggests high levels of chronic diseases and low utilization of preventive health services.^2^, ^3^ Health inequalities for PIs exist^5^ and with the introduction of western diets and the increased use of processed sugar and manufactured foods both in their homeland and in the US, there has been an increase in morbidity and mortality from infectious and chronic diseases, including dental caries.^2^, ^6^, ^7^, ^8^, ^9^, ^10^ Conventional strategies to reduce these changes have been unsuccessful.^11^, ^12^

Studies have suggested that PIs experience racism on all levels—personally mediated, internalized, and institutional—and that it leads to increasing stress and mistrust, leading to decreased access to services.^13^, ^14^ Studies have suggested that PIs experience several layers of barriers to health care. Two key barriers include racial discrimination and cultural differences from their home countries. A study conducted with healthcare providers, interpreters, and community members revealed that PIs experience racial discrimination on a regular basis from their healthcare service providers, in their local community and from other institutions such as public schools and the police department.^15^ The widespread racial discrimination currently faced by PIs, and the extensive history of war and exploitative treatment toward their home counties by the US, can cause PI patients to fear possible consequences of sharing health information with Western healthcare providers. Other barriers include difficulty communicating with providers, inadequate insurance coverage, and confusion about the healthcare system. For example, in several of the Pacific regions where patients migrate from, healthcare services are provided for free and there are no appointments needed because patients are taken on a walk‐in basis.^5^

## STATE‐LEVEL EFFORTS

Due to the significant lack of state‐level prevention and public health infrastructure in Hawaiʻi to address health disparities that disproportionately affect PIs, the initiatives developed thus far have been led by community organizations such as non‐profits, churches, and social service providers.^16^, ^17^ In a summary report^18^ that outlines 20 years of work by the Healthy Hawaiʻi Initiative, a multi‐sector, state‐wide public health collaborative, the following strategies were identified as integral in the successful targeting of health disparities among Native Hawaiian, Pacific Islander (PI), and Asian American populations: population‐based prevention focus, culturally based approaches, community‐clinical linkages, and the integration of prevention and treatment. The many barriers these patients face in accessing health care extends to dental care and there is a significant lack in prevention and public health infrastructure in Hawaiʻi to address oral health disparities that disproportionately affect PIs.^16^, ^19^

Unlike other US states, Hawaiʻi has only one centralized Department of Health with satellite district health offices and no department focused on oral health. Hawai'iʻs state Dental Health Division was eliminated in the late 2000s along with the state dental director position, ending school‐based screenings and any system for routine assessment of residents' oral health. There are currently no state‐wide oral health programs. In 2009 the state's Medicaid program also moved from comprehensive care to emergency‐only care for adults and has since remained at this level. Moreover, there is no community water fluoridation in Hawaiʻi except on military bases, with only 8.8% of the Hawaiʻi population served by community water systems receiving fluoridated water.^20^

## COMMUNITY‐LEVEL EFFORTS: KŌKUA KALIHI VALLEY COMPREHENSIVE FAMILY SERVICES

Kōkua Kalihi Valley Comprehensive Family Services (KKV) is a federally qualified health center (FQHC) in Honolulu, Hawaiʻi that serves the Kalihi Valley area. KKV was founded by community leaders in 1972, in response to inadequate healthcare services for Kalihi's population of primarily Asian and PIs (e.g., Filipino, Micronesian, Laotian, and Samoan), new‐immigrant and low‐income residents. Many are non‐English or limited English speakers and most live in the two largest public housing complexes in Hawaiʻi. There is a strong focus on KKV's motto, “neighbors being neighborly to neighbors,” with the organizational approach to clinical services centering around patient–provider relationships by implementing the social determinants of health (SODH) and culture‐based approaches. Since its founding, KKV has leveraged several strategies to develop culturally competent clinical services. These strategies include: collecting expertise from community members and empowering them as health educators and advocates, utilizing patient navigators and community health workers, and training KKV employees in cultural competence. That direct involvement of community members continues today. There has also been an intentional effort to hire individuals who live in the community and grow the organization's patient navigator and community health worker program. The latter has been considered one of the most successful strategies for improving healthcare access for patients of color and narrowing racial health disparities. Cultural competence is woven into various trainings for clinical and administrative staff and there are regular cross‐department meetings. Cultural immersion has also been an effective form of cultural competence training. Many of KKV's dental staff have engaged in cultural activities at the organization's 100‐acre nature preserve, Hoʻoulu ʻĀina, that provides land‐based and PI cultural programming. Staff have shared the experience improved their understanding of the community and their relationships with cultural workers at the organization.

Other culturally competent strategies have included extensive language accessibility through all communications materials for the main dialects of the community, including 26 different languages for all patient interactions (from front desk to provider), and increasing access through mobile clinics and school‐based programming. Previous studies have suggested that cultural competence trainings and programming have proven effective not only in strengthening patient relationships, but also in reducing racial and ethnic health disparities and improving overall quality of care.^17^, ^21^, ^22^

Dental care was the first service provided by KKV, primarily because community members went door‐to‐door asking Kalihi residents about their most significant health needs. Today the dental department has grown from used military trailers to a 12‐operatory main clinic that includes dental residency programs* that help to bring specialist‐level care to the community. It also includes a school‐based sealant program using mobile clinics, also known as the “Dental Bus”, that brings preventive oral health services to area local schools and ensures that all Kalihi youth have a dental home.^23^ The dental buses are a familiar and trusted symbol of KKV in the community, and frequently are requested by many children, schools, and events.

## COVID‐19 IMPACT AND PROGRAM ACTIVITIES

The COVID‐19 pandemic highlighted the inequities in the PI community in Kalihi.^24^ On March 16, 2020 the dental department followed national guidance and moved to emergency‐only dental care. In compliance, the department faced critical issues and needs in addressing the pandemic including the loss of jobs and income for the population and staff, ensuring access to food and supplies, and the immediate need to educate the community about preventing the spread of COVID‐19. In response to these needs, the dental department followed the lead of KKV's Elder Care program and designed a COVID‐19 outreach program with other KKV departments to stand in solidarity with a community that has experienced the impact of institutional racism and racial discrimination for generations. It was important for KKV to stand in solidarity with the community for multiple reasons including: (1) COVID‐19 would spread rapidly through close quarters in public housing units where many did not have space to quarantine or isolate; (2) many in the community did not understand concepts of quarantine, mask wearing, or other aspects of the virus; (3) many in the community feared testing because of generations of mistrust of the health system, as well as fear of testing positive and not having adequate space for quarantine, missing work, or getting evicted; (4) for years the community had looked to KKV for guidance; (5) KKV knew it needed to ensure equity of testing, care, and services that allowed people to weather the pandemic.

Dental staff joined other health center employees to focus on patients who were seniors, had mental or physical disabilities, or had comorbidities that qualified them as “high‐risk” for COVID‐19 symptoms. Staff called these patients to check‐in with them and screen for health status factors, emergency needs for food, medication, health care, socialization, and other supports for maintaining health and quality of life. These calls were opportunities to listen deeply to patients and an opportunity to build relationships, rather than cycling through a list of questions. This method of data collection is more familiar to the PI community and builds trust, rather than feeling extracted from—a common feeling from generations of mistrust of western health systems. Based on their status, dental staff made deliveries to the homes of patients and asked how they were feeling, while adhering to strict PPE protocol. Deliveries included oral hygiene supplies, medications, fresh produce, prepared healthy meals from KKV's cafe, technology support,^25^ and COVID‐19 kits that included informational materials, masks, soap, and hand sanitizer. The community's network of relationships helped KKV identify which families needed support, as community members would connect neighbors to the services. These deliveries and phone calls were more than just sharing resources; they were about building community, improving mental health, and helping people connect to the community.

Recognizing that many of the local mom and pop shops were frequent stops for the community, the dental staff also visited local businesses to check‐in with them and distribute public health messaging on COVID‐19. The Dental Bus, which is normally used for the school‐sealant program, was repurposed to distribute COVID‐19 kits and information at local public housing, and health center‐led COVID‐19 testing events. The familiarity of the Dental Bus in the community helped to attract community members to get tested, since there was great fear of being tested and the subsequent ramifications should they test positive (fear of being evicted, lack of space to isolate). At the main clinic, dental staff continued to provide emergency‐only dental care and patients were given a survey asking if they considered going to the emergency departments to stop the pain in their mouth or face if the dental clinic were not open.

The entire FQHC achieved the following results from March 2020 to July 2020:

- conducted over 800 outreach calls for health and food security,
- delivered over 2000 care packages and oral hygiene kits,
- visited 14 local businesses to support KKV's public health messaging campaign,
- community outreach increased COVID‐testing by 10‐fold,
- 796 unduplicated patients received emergency dental care, and
- of the 796 patients who received emergency care, over 47% of patients reported that if not seen, they considered going to the Emergency Room to stop the pain in their mouth or face.

## LESSONS LEARNED AND FUTURE DIRECTIONS

The pandemic allowed the dental department to expand beyond its traditional methods of providing culturally competent care to the PI community and enhance its SODH approach through more community engagement. The department also learned methods for data collection to better serve the needs of the community. More importantly, the dental department learned about the role it plays in potentially diverting costly dental‐related emergency room visits, as reflected by the 47% of patients who reported considering going to the emergency room to stop the pain in their mouth or face if not seen at KKV. The dental department conducted seven tele‐dentistry visits and learned that such visits are difficult without specialized intraoral camera equipment. However, tele‐dentistry may be useful for some post‐op visits and possibly preventive motivational interviewing check‐ins.

The community's responsiveness to KKV's COVID education and outreach efforts may reflect its value on social connections and strong relationships, a core value in PI culture,^26^ which the organization and dental staff have developed with patients, as well as the power of culturally appropriate services. This suggests that KKV's best strategy for countering racial prejudice, systemic racism, and the oppression of minority groups, in particular the PI immigrants who make up a majority of KKV's patient population, may be through prioritizing meaningful relationship building in every patient interaction.^27^ Examples include (1) capitalizing on relationships built with community and religious leaders, as well as community members who are revered and known as elders or “super aunties” who provide guidance to the community, and getting these respected individuals to spread the message on the importance and value of oral health and bridge the oral health literacy gap, to seek routine care and not only seeking care when they are in pain; (2) designing a health system that is accommodating to PI culture of walk‐in appointments; (3) using mobile clinics to bring care to them. This improves racial inequities in community health, and supports the larger movement toward improved quality of care, oral health outcomes, and patient satisfaction and retention.^28^

Future work for the dental department includes identifying opportunities to incorporate these lessons in its school‐based sealant program, coordination with other KKV programs, oral health education and prevention, and data collection and evaluation regarding community oral health disparities. This includes identifying opportunities to check‐in with patients since this pandemic outreach program has revealed the need for social connections, a core value in many PI cultures. For example, screening questions for COVID‐19 can be transformed to ask about the general well‐being of individuals in ways that deepen the dentist‐patient relationship and build trust which could increase patient adherence and loyalty, and lead to better therapeutic results and anxiety management.^29^ Moreover, checking in with individuals and building relationships can be incorporated to oral health education and prevention and follow‐ups with high‐risk individuals identified in the school‐based programs, young children in WIC programs, and those receiving well‐child checks.^30^ Strong relationships could make such oral health education and prevention a powerful strategy to prevent oral diseases from a young age.

## Footnotes

* Today there is only an Advanced Education in General Dentistry (AEGD) program. Previously, there were also residencies in pediatric dentistry and dental public health.
